# Validation and application of OCT tissue attenuation index for the detection of neointimal foam cells

**DOI:** 10.1007/s10554-020-01956-9

**Published:** 2020-08-06

**Authors:** Philipp Nicol, Petra Hoppman, Kristina Euller, Erion Xhepa, Tobias Lenz, Himanshu Rai, Hiroyuki Jinnouchi, Anna Bulin, Maria Isabel Castellanos, Anna Lena Lahmann, Tobias Koppara, Adnan Kastrati, Michael Joner

**Affiliations:** 1 Klinik für Herz- und Kreislauferkrankungen, Deutsches Herzzentrum München, Technische Universität München, Lazarettstrasse, 36, Munich, Germany; 2 Klinik und Poliklinik für Innere Medizin I, Klinikum rechts der Isar, Technische Universität München, Munich, Germany; 3 DZHK (German Centre for Cardiovascular Research), Partner Site Munich Heart Alliance, Munich, Germany; 4 CVpath Institute Inc., a Non-profit Organization in Gaithersburg, Gaithersburg, MD USA

**Keywords:** Intravascular imaging, Optical coherence tomography, Neoatherosclerosis, Translational research, Angioplasty

## Abstract

Neointimal infiltration with foamy macrophages is recognized as an early and important sign of de-novo atherosclerosis after stent implantation (neoatherosclerosis). Recent histopathological studies have proven that automated quantification of signal attenuation using intravascular optical coherence tomography (OCT) imaging allows for sensitive identification of macrophages in native atherosclerotic disease. Whether this is true for neointimal foam cells in the setting of neoatherosclerosis remains unknown. Autopsy samples of stented coronary arteries (n = 13 cases) were evaluated by histology and OCT. After co-registration with histology, the attenuation rate of emitted laser light was measured in regions with and without neointimal foamy macrophages relative to its peak intensity at the blood-tissue interface. Attenuation index was subsequently determined as slope of a regression curve fitted to individual data points. Receiver operating curve (ROC) analysis was used to establish an optimal cut-off for detecting foamy macrophages in homogenous and non-homogenous neointima, respectively. Finally, the tissue attenuation index was applied to confirm or exclude the presence of neointimal foamy macrophages in symptomatic patients presenting with in-stent restenosis and undergoing intravascular OCT imaging (n = 29 cases). Tissue attenuation index derived from post-mortem samples differed significantly between histologically confirmed regions with and without neointimal foamy macrophages (− 1.23 ± 1.42 vs. − 0.52 ± 1.79, p < 0.05). ROC analysis was able to distinguish neointima with foamy macrophage infiltration from neointima without (93% sensitivity, 73% specificity, cut-off − 0.79, AUC 0.87 for homogenous neointima and 40% sensitivity, 95% specificity, cut-off − 1.93, AUC 0.69 for non-homogenous neointima). In symptomatic patients presenting with in-stent restenosis after stent implantation and undergoing intravascular imaging with OCT, neointimal foamy macrophages were detected in 34.2% of homogenous and 43.6% of non-homogenous neointimal ROI’s evaluated. OCT-derived and histopathologically validated tissue attenuation index enables identification of neointimal foamy macrophages in stented coronary arteries. Such image-based post-processing software algorithm may help discern and triage subjects at increased risk for device-related events.

**Electronic supplementary material:**

The online version of this article (10.1007/s10554-020-01956-9) contains supplementary material, which is available to authorized users.

## Introduction

Identification of native atherosclerotic plaques with a high probability of rupture (“vulnerable” or “unstable plaques”) is essential for detecting patients at risk for acute coronary syndromes (ACS). Post-mortem autopsy studies demonstrated that vulnerable lesions are characterized by large lipid pools, thin fibrous caps and high content of macrophages [1]. High-resolution intravascular imaging with optical coherence tomography (OCT) is able to visualize key features of atherosclerotic plaque development [2]. Previous studies have shown that macrophages can be detected by OCT either using a visual inspection method (bright signal with typical light attenuation) [3] or automated quantification of OCT signal attenuation [4–6].

Whether this is also true for neointimal infiltration with foamy macrophages, a key parameter of early in-stent atherosclerosis (neoatherosclerosis), remains unknown to date. Neoatherosclerosis has been recognized as accelerated manifestation of atherosclerosis after implantation of bare metal (BMS) and drug-eluting stents (DES) [7, 8].

By histology, early stage neoatherosclerosis is characterized by clusters of foamy macrophages within the peri-strut area or along the luminal neointima. These can progress to form fibroatheroma and develop necrotic cores containing acellular debris and free cholesterol. Lately, neoatherosclerosis with rupture of thin-cap fibroatheroma (TCFA) has been recognized as the predominant cause in patients presenting with very late stent thrombosis [9], where foamy macrophage infiltration of the fibrous cap was identified as important surrogate of neointimal plaque vulnerability [10]. Neointimal foamy macrophages cause a characteristic imaging pattern during intravascular imaging with OCT [11, 12]. However, histopathologically validated studies investigating OCT-derived parameters for sensitive detection of early stage neoatherosclerosis are lacking to date. Reliable detection of neointimal foam cells may enable identification of patients at risk for stent-related adverse events including in-stent restenosis and stent-thrombosis. The aim of this study was therefore to investigate whether tissue attenuation differs between regions with and without neointimal foam cell infiltration and, whether tissue attenuation index could reliably identify patients with neointimal foamy macrophage infiltration as an early sign of neoatherosclerosis.

## Materials and methods

**Fig. 1:**
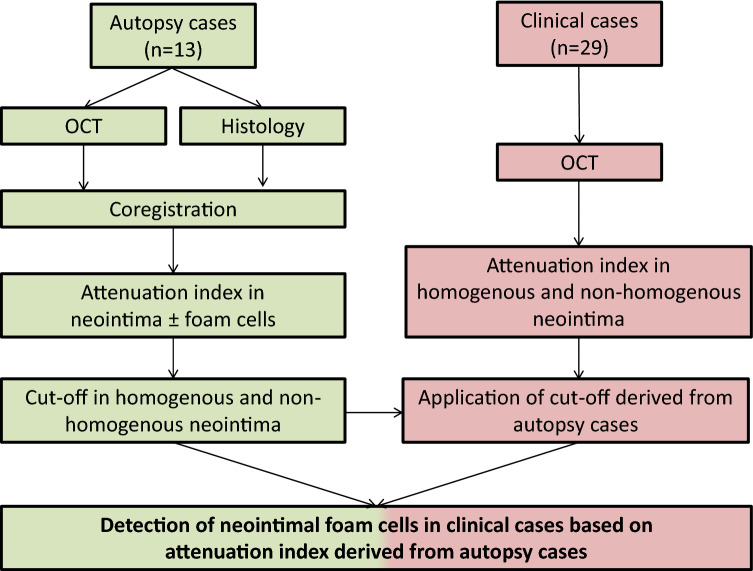
Study flow

Please see Fig. 1 for overview of study flow.

### Human autopsy samples and ex vivo OCT imaging

**Fig. 2:**
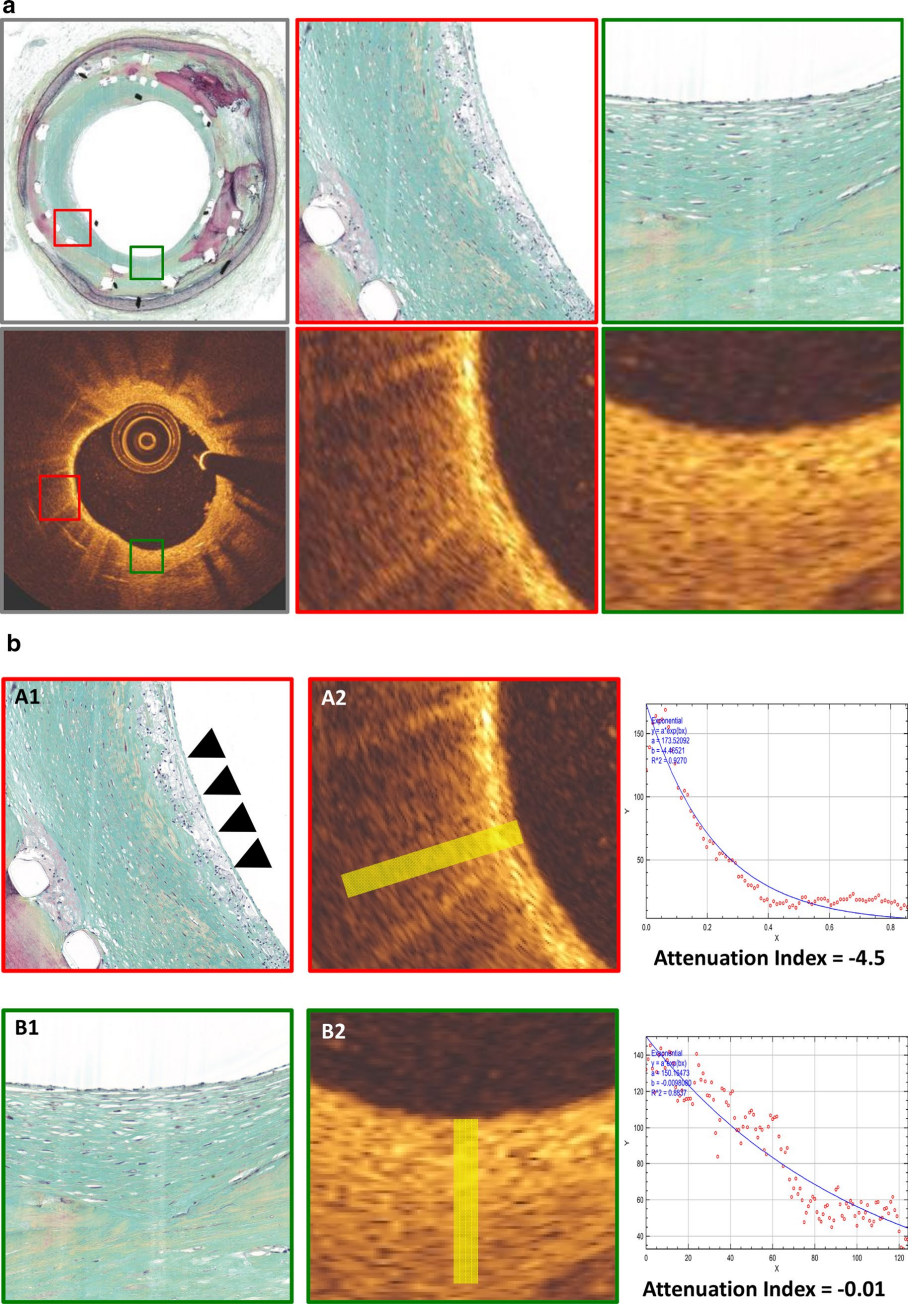
**a** Healthy and diseased neointima in a coronary autopsy sample. Histopathology (Movat pentachrome staining, upper row) and corresponding OCT (lower row) from the RCA of a 64-year-old male dying of non-cardiac death. A DES (Driver 4 × 14 mm) was implanted in the RCA over 5 years ago. Magnified area (red frame) shows diseased neointima with superficial foam cell infiltration while healthy neointima is magnified in green frame. **b** Tissue attenuation index in neointima with and without foam cell infiltration. Histopathology (left, A1 and B2) and intravascular OCT (middle, A2 and B2) from **a**. Upper panel shows neointima infiltrated with neointimal foam cells (arrows) while healthy neointima without foam cell infiltration is observed in the lower panel. Right: Graphical plot of attenuation index derived from ROI (yellow box in A2 and B2) showing distinctly different attenuation indices

Autopsy samples of stented coronary arteries were acquired from medical examiners (n = 13 cases). After careful preparation, vessels were wired with a 0.014-inch guide wire over which the OCT catheter (2.7-F, St. Jude Medical, St. Paul, Minnesota) was advanced. An imaging pullback was performed from the distal to the proximal arterial segments while simultaneously flushing the vessel with contrast to improve imaging quality (pullback speed 5 mm/s = 120 frames/s). Afterwards, arteries were segmented at 3 mm intervals and stained with haematoxylin–eosin (H&E) as well as Movat pentachrome. Presence of neointimal foamy macrophages was classified on nominal scale as either present or absent. Co-registration of OCT frames and histological sections of human autopsy samples was achieved as previously described [12]. To accurately identify areas of neointimal foam cell infiltration, co-registered OCT frames were first divided into four quadrants and classified as either homogenous (uniform light reflection without localized areas of stronger or weaker backscattering properties) or non-homogenous (focal variation of the backscattering pattern including patterns commonly described as “heterogenous” and “layered”). Subsequently, each quadrant was assigned a nominal score for the presence or absence of neointimal foam cells after confirmation with co-registered histological sections. OCT tissue attenuation index was measured in foam-cell positive regions identified by histology while foam-cell negative regions served as control (see Fig. 2a, b).

### Clinical study population

Symptomatic patients after stent implantation and presenting with in-stent restenosis following invasive diagnostic work-up using intravascular imaging by OCT were retrospectively included (n = 29 cases). For baseline data of patients and morphometric measurements derived from OCT, see Table 1. OCT imaging of in-stent restenosis was performed according to current guidelines [2, 13] with commercially available OCT systems (Dragonfly DF-OCT-catheter combined with the C7-XRTM imaging system; LightLab Imaging Inc., Westford MA, USA).

**Table 1:** Baseline data of patients with ISR

n	29	
Sex (n)		
Male	24	
Female	5	
Age (years)	68 (± 12.5)	
Duration of FU (days)	925 (± 1683)	
Culprit vessel (n)		
LAD	17	
LCx	5	
RCA	7	
Lesion length (n)		
< 10 mm	5	
10–20 mm	16	
> 20 mm	8	
Type of stent (n)		
DES	24:	
	1st Gen. DES	9
	2nd Gen. DES	15
BRS	2	
BMS	3	
Morphometry (OCT)		
Lumen area (mm^2^)	4.91 (3.48–6.565)	
Lumen diameter (mm)	2.50 (2.10–2.88)	
Stent area (mm^2^)	7.43 (6.09–9.37)	
Neointimal area (mm^2^)	2.28 (1.39–3.65)	
Neointimal thickness (mm)	0.25 (0.13–0.43)	
Covered struts (%)	94	
Malapposed struts (%)	1	
Clinical presentation (n)		
STEMI	1	
NSTEMI	2	
Unstable AP	2	
Stable AP	13	
Silent Ischemia	11	

### OCT analysis and measurement of attenuation index

Offline analysis of OCT pullbacks was performed using ImageJ (Wayne Rasband, National Institute of Health, USA). All frames were independently assessed by experienced investigators (KE for autopsy cases and PN and PH for clinical cases). OCT frames were systematically evaluated at 1-mm increments starting at the first frame in which stent struts were visible along the entire vascular circumference.

For autopsy cases, attenuation index was measured in histologically confirmed regions with and without neointimal foamy macrophages that were classified as homogenous or non-homogenous in tissue backscattering pattern.

OCT frames of clinical cases were divided into four quadrant and classified as homogenous or non-homogenous in tissue backscattering pattern. Subsequently, tissue attenuation index was measured in all four quadrants: For each quadrant, four regions of interest (ROI), evenly distributed along the circumference of the quadrant were chosen for measurement of tissue attenuation index. As general rule, four regions of interest were measured, with the exception of guidewire artefact obscuring the underlying tissue, presence of side-branches and insufficient neointimal thickening to derive tissue attenuation index, resulting in three regions of interest per quadrant.

For calculation of tissue attenuation index, OCT pullbacks were first transformed into 8-bit grey scale images. Signal intensities were calibrated using grey scale values of the lumen as well as the brightest pixel of the guide wire and set as reference in each analysable frame (the brightest level of the guide wire being set as maximum and the darkest level of the lumen as minimum). For calibration of dimensions, the diameter of the OCT catheter was measured and set as a reference (2.7F = 0.9 mm). To account for varying neointimal thickness (mean neointimal thickness in autopsy cases: 0.33 mm ± 0.16), ROIs were manually traced at a maximum distance of 400 μm from the endoluminal surface. Signal intensities for each pixel of an ROI (luminal surface to the strut edge) were then graphically plotted as a function of distance from the endoluminal surface using ImageJ. Attenuation index was subsequently determined as slope of a regression curve fitted to individual data points (decrease of signal intensities as a function of distance from the endoluminal surface), which resulted in a single attenuation index for each ROI [14].

To investigate whether an eccentric position of the OCT imaging catheter had an influence on the derived attenuation index, frames were also divided according to catheter position (central versus decentral): for defining central or decentral catheter position, the luminal space was divided by drawing a circle with a diameter representing 50% of the total vessel diameter. A central position was defined if the imaging catheter (IC) was found to be within that circle. Position of the imaging catheter outside that circle was defined as decentral (or eccentric).

### Statistical analysis

Continuous data were checked for normality of distribution using Wilk-Shapiro test and expressed as means with standard deviation in case of normal distribution and median with interquartile range in case of non-parametric distribution. To account for the clustered nature of the data, a generalized linear mixed model was conducted for the analysis of OCT data. Regression analysis was performed according to iterative curve fitting algorithms using ImageJ software package (Version 2.0). F-test along with goodness-of-fit statistics (R^2^) were applied to find the most suitable regression curve to fit individual data sets. Logistic regression with receiver operating characteristics (ROC) analysis was used to investigate diagnostic accuracy of mean attenuation index for detection of neointimal foam cells from autopsy cases. Youden’s rule was applied to detect the optimal cut-off points. Intra- and interobserver variability of attenuation measurements was calculated with intra- and interobserver correlation coefficients (ICCs). Analysis was carried out using JMP Version 13.0 software (SAS, Cary, NC). P < 0.05 was considered statistically significant.

## Results

### Human autopsy samples of stented coronary arteries

A total of 19 coronary segments from 13 autopsy cases were investigated through standard histopathological processing as described above. Please see Supplemental Table 1 for baseline data of autopsy cases. A total of 45 frames with 318 regions of interest (ROIs) were analysed consisting of homogenous neointima (n = 150 ROIs) and non-homogenous neointima (n = 168) with neointimal foam cells detected in 30% of homogenous (45/150) and 60.1% of non-homogenous neointima (101/168), respectively (see Table 2; Fig. 2a, b).

**Table 2:** Overview of autopsy and clinical cases

	Autopsy cases		Clinical cases
Cases (n)	13		29
Frames (n)	45		348
Regions of interest (n)	318		1483
Homogeneous ROIs (%)	47.2 (150/318)		64.4 (955/1483)
	Foam cells +	30 (45/150)	n.a
	Foam cells –	70 (105/150)	n.a
Non-homogenous ROIs (%)	52.8 (168/318)		35.6 (528/1483)
	Foam cells +	60.1 (101/168)	n.a
	Foam cells –	39.9 (67/168)	n.a

### Measurement of attenuation index in human autopsy samples of stented coronary arteries

Post-mortem cross-sectional OCT frames were carefully co-registered with corresponding histopathological sections using anatomic landmarks and distance allocation (Fig. 2a). Measurement of tissue attenuation index was performed in n = 146 ROIs showing neointimal foam cells (above stent struts n = 74, between stent struts n = 72) while areas without neointimal foam cell infiltration served as control (n = 172, above stent struts n = 85, between stent struts = 87, Fig. 2b). Mean attenuation index differed significantly between foam-cell positive and foam cell-negative neointima with a mean attenuation index of − 1.23 (± 1.42) in the presence of neointimal foam cells and a mean attenuation index of − 0.52 (± 1.79) in the absence of neointimal foam cells (p < 0.05; see Table 3). When further divided into homogenous and non-homogenous neointima, mean tissue attenuation index in homogenous neointima with foam cells was − 1.34 (± 0.43) compared to − 0.50 (± 2.1) in homogenous neointima without foam cells. In non-homogenous neointima, mean attenuation index was − 1.47 (± 1.22) and − 0.48 (± 0.54), respectively.

**Table 3:** Mean tissue attenuation index in autopsy and clinical cases

Autopsy (n = 13)	Tissue attenuation index (± SD)	Neointima (+) Foam cells		Neointima (−) Foam cells	
		− 1.23 (1.42)		− 0.52 (1.79)	
	p	< 0.05			
	Tissue attenuation index (± SD)	Non-homogenous		Homogenous	
		+ Foam cells	− Foam cells	+ Foam cells	− Foam cells
		− 1.49 (1.22)	− 0.48 (0.54)	− 1.34 (0.43)	− 0.50 (2.1)
Clinical (n = 29)	Tissue attenuation index (± SD)	Non-homogenous		Homogenous	
		− 1.53 (1.74)		− 0.45 (3.2)	

**Fig. 3:**
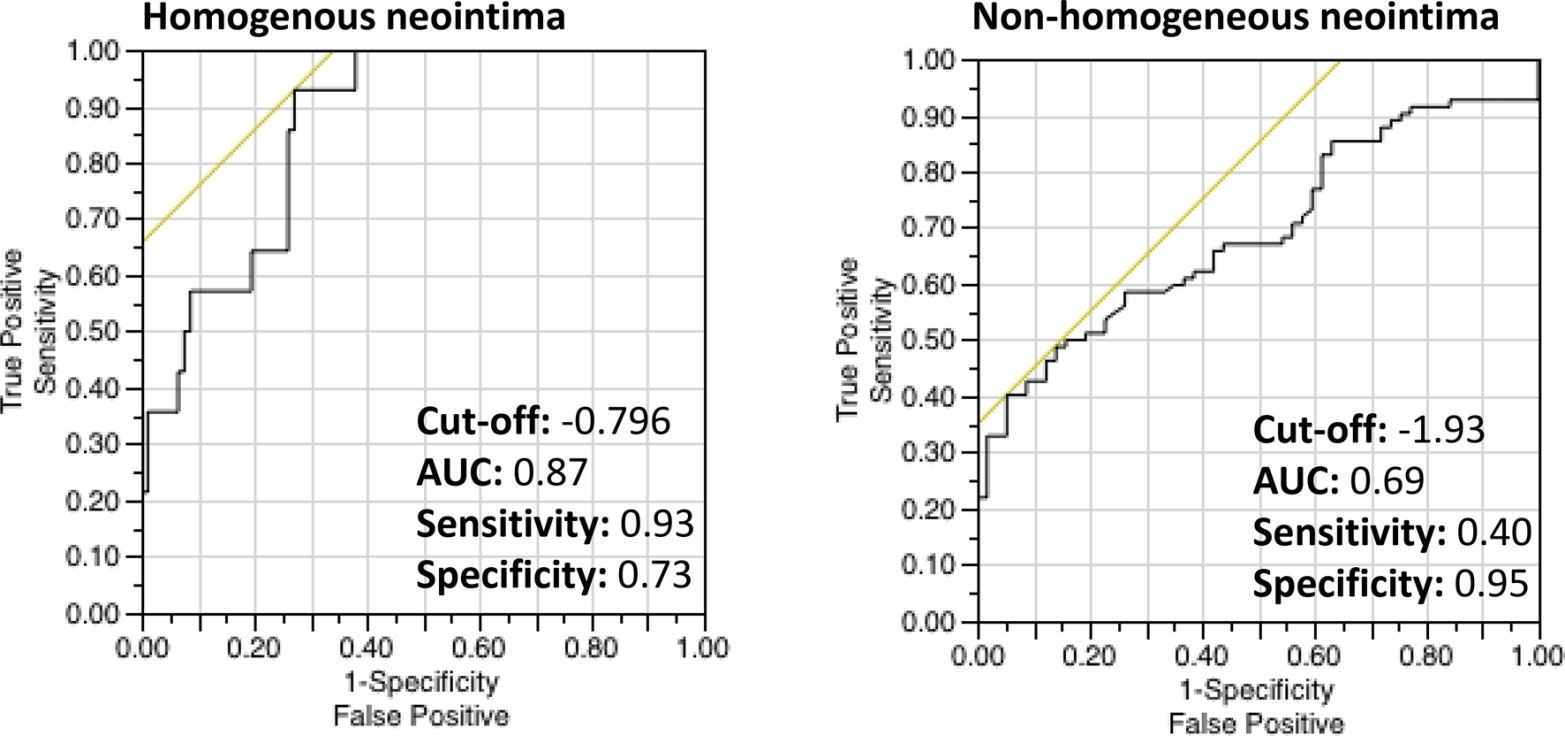
ROC analysis of tissue attenuation index for detection of neointimal foamy macrophages

Accuracy of this histopathological derived tissue attenuation index for detection of neointimal foamy macrophages was analysed using receiver-operating curves (ROC), with individual cross-sectional OCT frames being classified into groups with homogenous vs. non-homogenous tissue backscattering [2, 15]. ROC analysis showed high sensitivity for confirming the presence of neointimal foam cells in homogenous neointima (sensitivity 93%, specificity 73% using cut-off − 0.79, AUC 0.87). In the case of non-homogenous neointima, tissue attenuation index revealed moderate discriminative power with sensitivity and specificity of 40% and 95%, respectively (cut-off -1.93, AUC 0.69, see Table 4 and Fig. 3). Variability in attenuation measurements showed very good agreement regarding intra- and interclass correlation coefficient (0.95 and 0.91, respectively). Position of the imaging catheter in the vessel lumen (central vs. decentral or “eccentric” position) did not have a significant influence on derived mean attenuation index (see Table 5).

**Table 4:** ROC analysis of attenuation index for detection of neointimal foam cells

	Cut-off	AUC	Sensitivity	Specificity	PPV	NPV
Homogenous Neointima + Foam cells	− 0.79	0.87	0.93	0.73	0.57	0.44
Non-homogenous Neointima + Foam cells	− 1.93	0.69	0.40	0.95	0.30	0.70

**Table 5:** Mean attenuation index in frames with central vs. decentral position of imaging catheter

	Central (n = 150)	Decentral (n = 198)	p-value
Homogenous	− 0.72 (± 1.92)	− 0.32 (± 3.96)	0.61
Non-homogenous	− 1.54 (± 1.86)	− 1.68 (± 1.83)	0.42

### Application of attenuation index for detection of neointimal foam cells in clinical cases of in-stent restenosis

Tissue attenuation index was used to retrospectively analyse OCT pullbacks from symptomatic patients presenting with in-stent restenosis (ISR) after stent implantation and undergoing intravascular OCT imaging (n = 29 cases).

**Fig. 4:**
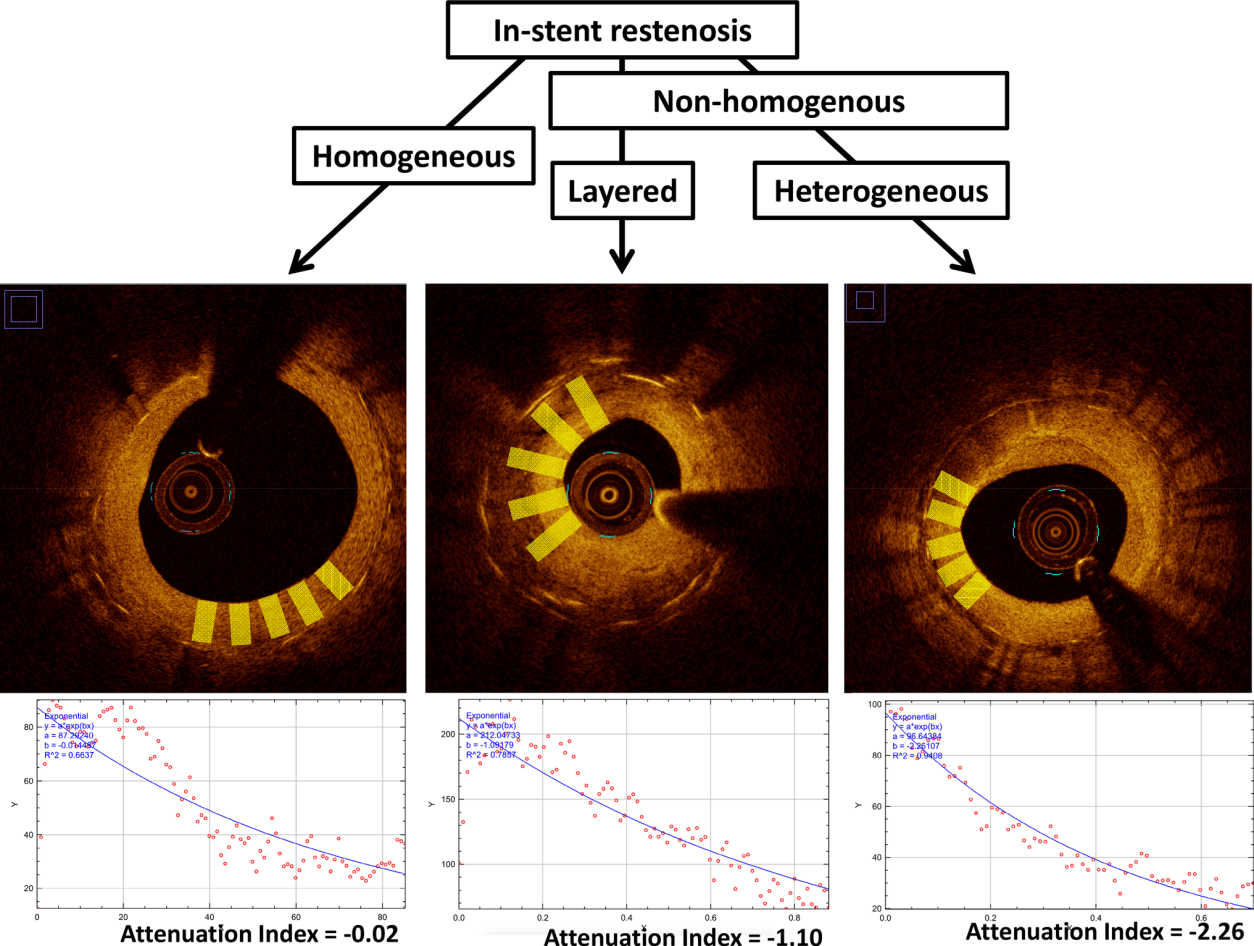
Neointimal patterns of in-stent restenosis. **a** Homogenous vs. non-homogenous neointima in clinical cases of in-stent restenosis with **b** derived mean attenuation index

**Fig. 5:**
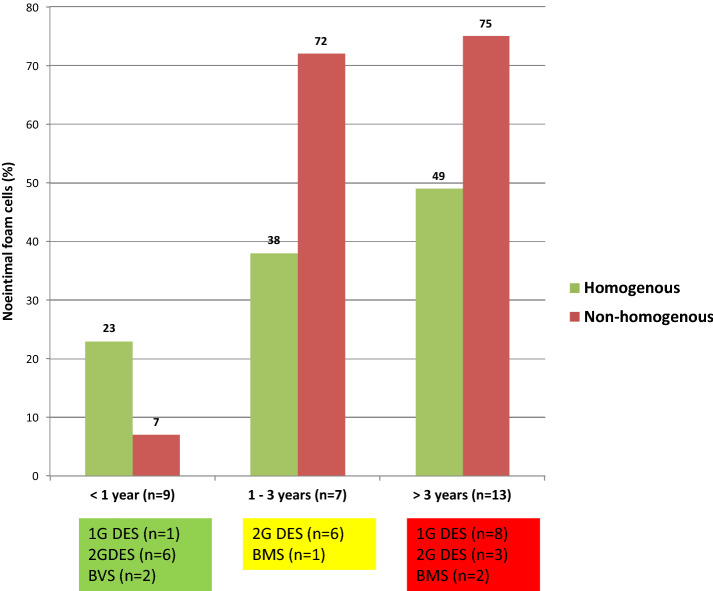
Time-dependent formation of neointimal foam cells in clinical cases of in-stent restenosis. Neointimal foam cells detected by attenuation index in stents less than 1 year after implantation (green box), 1–3 years (yellow box) and over 3 years (red box) for homogenous (green column) and non-homogenous neointima (red column)

A total of 348 frames with 1483 regions of interest were investigated, consisting of various types of neointima (homogenous, n = 955; non-homogenous, n = 528, see Table 2 and Fig. 4). Mean tissue attenuation index was − 0.47 (± 0.76) in homogenous neointimal quadrants and − 1.15 (± 1.11) in non-homogenous quadrants. In a second step, the cut-off derived from ROC analysis in autopsy samples was applied to assess neointimal foam cell infiltration in clinical cases of in-stent restenosis, showing presence of foam cells in 34.2% of homogenous neointimal ROIs (327/955) and 43.6% of non-homogenous neointimal ROIs (230/528; Table 6). Tissue attenuation index also showed a time-dependent incidence of neointimal foam cell when clinical cases were divided upon duration of stent implantation (less than 1 year, 1–3 years and above 3 years (Fig. 5).

**Table 6:** Detection of neointimal foam cells in autopsy and clinical cases

		Homogenous (n = 150)	Non-homogenous (n = 168)
Autopsy (n = 13)	+ Foam cells (%)	30 (45/150)	60.1 (101/168)
	− Foam cells (%)	70 (105/150)	39.9 (67/168)

		Homogenous (n = 955)	Non-homogenous (n = 528)
Clinical (n = 29)	+ Foam cells (%)	34.2 (327/955)	43.6 (230/528)
	− Foam cells (%)	66.8 ( 628/955)	56.4 (298/528)

## Discussion

The objectives of the presented study were twofold. Firstly, we sought to perform histopathological validation of OCT-based tissue attenuation index for the detection of foamy macrophages in human autopsy samples; secondly, we aimed to apply the novel OCT image-based post-processing algorithm in clinical cases of in-stent restenosis to identify patients with foamy macrophage infiltration as early sign of neoatherosclerosis. In this regard, the salient findings of our study are:

(i) Application of a novel OCT image-based post-processing algorithm to derive tissue attenuation index is feasible and aids in detecting neointimal foamy macrophages in human autopsy samples of stented coronary arteries.
(ii) Foam cell infiltration of neointimal tissue caused significantly increased tissue attenuation index compared to neointima without foamy macrophages, where ROC curve analysis revealed the best cut-off for detecting neointimal foamy macrophages with − 0.79 in homogenous and − 1.93 in non-homogenous tissue backscattering quadrants.
(iii) Application of the previously established cut-off for detecting neointimal macrophage infiltration in 29 patients presenting with in-stent restenosis revealed a significant proportion (34.2% with homogenous neointima and 43.6% with non-homogenous neointima) of cases with early neoatherosclerotic change.

### Attenuation index for identification of foamy macrophages

Contemporary intravascular imaging with high-resolution OCT is based on the principle that light from a low-coherence source is split into two paths by a coupler directing it along two different arms of an interferometer. One arm is designated as reference arm, and the other as sample arm. The final tomographic OCT image is derived from interference between reference and sample arms followed by specific software data post-processing. During tissue transition, near-infrared light is subject to backscattering and attenuation secondary to differential refractive indices of tissue components resulting in specific optical densities [16, 17]. Attenuation is caused by backscattering of the near-infrared light beam at the interface between materials of different refractive indices and has been utilized to identify macrophages in native atherosclerosis [4].

Tearney et al. correlated OCT with histologically-confirmed infiltration of macrophages in lipid-rich plaques obtained post-mortem and used normalized standard deviation (NSD) of the OCT signal to identify areas with significant macrophage infiltration [5]. In a similar study, tissue quantification was performed using NSD, OCT signal attenuation and a specific granulometry index to investigate detection of inflamed regions in atherosclerotic plaques. Using a two-step algorithm (applying NSD first, followed by granulometry index) delivered good sensitivity and specificity in identifying inflamed ROIs, making this approach however also rather impractical for daily practice [6].

Interpretation of OCT imaging is subject to high inter-observer variability and most descriptive terms used in clinical practice to date show little correlation to histopathology [12]. Previous work has further provided evidence that “bright spots” on OCT (commonly thought to represent macrophages) are caused by a sudden change in the refractory index at interfaces between plaque components and eventually constitute a variety of atherosclerotic plaque components, such as fibrous or calcific tissue [18]. Consequently, novel automated algorithms to detect specific plaque features associated with progression of atherosclerosis are highly warranted.

In a previous work of our group, we showed that quantification of OCT-based grey scale-signal intensities (GSI) enables differentiation of histologically confirmed mature and immature neointimal tissue types [19]. Yet, reliable detection of individual tissue components has not been achieved with contemporary intravascular imaging methodologies, to date. Neoatherosclerosis with neointimal foam cell infiltration as its earliest sign contributes to long-term failure of stented coronary segments. In a large registry investigating very-late stent thrombosis, neointimal plaque rupture was observed as the underlying aetiology in 31.3% of patients [9]. Infiltration with neointimal foamy macrophages was significantly higher in ruptured compared to stable plaques [10] and may consequently serve as surrogate for plaque vulnerability in the setting of neoatherosclerosis.

In patients with in-stent restenosis (ISR), a recent work investigating neointimal characteristics found features of neoatherosclerosis in 30.8% of lesions [20]. Therefore, reliable detection of neointimal foam cells could enable identification of patients at risk for future device-related events. Currently, the evidence regarding feasibility of OCT to reliably detect neointimal infiltration with foamy macrophages is limited.

In our work, histological evaluation of post-mortem coronary arteries and careful co-registration with intravascular OCT enabled accurate identification of vascular regions with foamy macrophage infiltration. However, it is important to note that key settings of this post-processing algorithm such as calibration and measurement of attenuation index are bound to individual device configurations and procedural factors (such as coronary hemodynamics and clearance of blood). As a result, findings of the current study may not be translatable to different imaging settings, where individual calibration is needed to derive appropriate cut-off values for the detection of neointimal foam cells. Yet, these are limitations inherent to most contemporary attempts to standardize imaging-based tissue characterization. To overcome these limitations, more sophisticated and advanced approaches involving artificial intelligence algorithms are ultimately required.

### Additional sources of signal attenuation

Our work focused on the detection of neointimal foamy macrophages, which were shown to cause significant light attenuation [21, 22]. In a previous autopsy study to examine differential diagnosis of most relevant OCT imaging features, we were able to show that significant attenuation of OCT signal close to the endoluminal surface can be caused by various tissue components such as elastic fibres and calcification [12]. Signal attenuation in the peri-strut area on the other hand, was associated with neovascularization [19], which implies that attenuation of near-infrared light is the result of distinct optical properties arising from local tissue composition and spatial relationship to the endoluminal vascular surface. Since we could not account for the majority of these factors resulting in signal attenuation, overlap with previously mentioned differential diagnosis may be possible [12]. These limitations are further mirrored by the fact that detection of neointimal foam cells was strongly dependent on underlying tissue quality, where application of attenuation index in non-homogenous neointimal tissue revealed rather low sensitivity (40%) and may therefore not yield high enough diagnostic accuracy to detect neointimal foam cells. However, very high specificity of 95% in this setting may enable exclusion of neointimal foam cells. When investigating homogenous neointima, tissue attenuation index showed very high sensitivity (93%) and acceptable specificity (73%), permitting confirmation or exclusion of foamy macrophages.

Application of the tissue attenuation index in a selected set of clinical cases with in-stent restenosis allowed detection of foamy macrophages in 34.2% of homogenous and 43.6% of non-homogenous neointima, respectively. Given the above-mentioned limitations, the percentage of neointimal quadrants with foam cell infiltration may rather be over-than underestimated. Interestingly, the frequency of quadrants with neointimal foam cell infiltration was similar between autopsy and clinical cases (30% vs. 34.2%) when limited to homogenous neointima (Table 3). Although the number of individuals investigated in the autopsy study is too small to draw definite conclusions, this finding may further confirm transferability and translational aspects of our findings.

In conclusion, calculation of tissue attenuation index may be considered a helpful and easy applicable supportive tool in the clinical setting when using intravascular optical coherence tomography (OCT) for identification of foam cell infiltration as early sign of neoatherosclerosis. Whether identification of foam cells has therapeutic implications and could be used to monitor treatment response in patients with progression of atherosclerosis has to be evaluated in dedicated trials.

## Limitations

This is a translational study with three major limitations: first, the presented findings are based on use of ex-vivo post-mortem OCT, which might differ from in-vivo imaging (e.g. regarding optical properties). This could impede the transferability towards application in large-scale clinical studies. Second, data were obtained from a small set of subjects thus resulting in a limited sample size and third, acquired only retrospectively.

## Electronic supplementary material

Below is the link to the electronic supplementary material.

Supplementary file1 (DOC 17 kb)
