# Supplementary material for: Validation and application of OCT tissue attenuation index for the detection of neointimal foam cells
Source: Int J Cardiovasc Imaging. 2020 Aug 6;37(1):25–35. doi: 10.1007/s10554-020-01956-9 (PMC7878214; doi:10.1007/s10554-020-01956-9)
Supplement: Supplementary file 1 — Supplementary file1 (DOC 17 kb) [file 10554_2020_1956_MOESM1_ESM.doc]

| **Case** | **vessel** | **Device** | **Duration** | **Age** | **Gender** | **DM** | **HT** | **HL** | **Smoking** | **Cause of death** |
| --- | --- | --- | --- | --- | --- | --- | --- | --- | --- | --- |
| 1 | pLAD | Cypher 4*18mm | >1000 | 48 | M |  | yes |  |  | non-cardiac death |
| 2 | LAD | 2.5 x 12 mm Driver | >365 | 51 | M |  | yes |  |  | non-cardiac death |
| 3 | pRCA | Palmaz-Schatz 3.5x14 mm | > 4300 | 72 | F |  | yes |  |  | non-cardiac death |
| 4 | dRCA | Endeavor 3x23 mm | > 720 | 62 | M |  | yes |  |  | Stent related death |
| 5 | pRCA | Vision 3x18 mm | 180 | 37 | M | yes | yes | yes | yes | non-cardiac death |
| 6 | pLAD | 3 x 35 mm Cypher | > 1000 | 62 | M | yes | yes | yes |  | non-cardiac death |
|  | mLAD | Multilink Zeta 3.5x22 mm | > 1000 | 62 | M | yes | yes | yes |  | non-cardiac death |
| 7 | pLAD | Vision 3x12 mm | > 720 | 53 | M | yes | yes |  |  | Cardiac death |
|  | pRCA | Vision 3x12 mm | > 720 | 53 | M | yes | yes |  |  | Cardiac death |
| 8 | mLAD | Taxus 3x23 mm | > 1000 | 49 | M |  |  |  |  | Stent related death |
|  | mRCA | 4 x 16 mm Cypher stent | > 1000 | 49 | M |  |  |  |  | Stent related death |
| 9 | mRCA | Xience 3x28 mm | 52 | 86 | F | yes | yes | yes |  | non-cardiac death |
| 10 | mLAD | Xience 3x12 mm | 365 | 75 | M |  | yes | yes | yes | non-cardiac death |
| 11 | mLAD | Resolute 2x25 mm | 238 | 55 | F |  |  |  |  | non-cardiac death |
|  | pLAD | Resolute 2x30 mm | 238 | 55 | F |  |  |  |  | non-cardiac death |
| 12 | pLAD | BX Velocity 3x17 mm+2*13mm | > 1000 | 48 | M |  |  |  |  | non-cardiac death |
| 13 | dRCA | Driver 4x14 mm | > 1800 | 64 | M |  | yes | yes | yes | non-cardiac death |
|  | mRCA | Multilink/Xience/Multilink overl. Stents  (total 4*30mm) | > 5800/1800 | 64 | M |  | yes | yes | yes | non-cardiac death |

**Supplemental Tab. 1:** Baseline data of autopsy cases
